# Investigating the molecular mechanisms of deltamethrin resistance in *Musca domestica* populations from Saudi Arabia

**DOI:** 10.1186/s13071-025-06668-4

**Published:** 2025-02-16

**Authors:** Ali A. Alzabib, Ali S. Al-Sarar, Yasser Abobakr, Amgad A. Saleh

**Affiliations:** 1 Department of Plant Protection, College of Food and Agriculture Sciences, King Saud University, P.O. Box 2460, 11451 Riyadh, Saudi Arabia; 2 Department of Animal Pests, Plant Protection Research Institute, Agricultural Research Center, Alexandria, 21616 Egypt; 3 Agricultural Genetic Engineering Research Institute, Agriculture Research Center, Giza, 12619 Egypt

**Keywords:** *Musca domestica*, Deltamethrin, Knockdown resistance, *kdr* mutation, *Vssc*, *CYP6D1v1*, Saudi Arabia

## Abstract

**Background:**

The house fly, *Musca domestica* L., is a global insect pest that poses serious health risks by carrying pathogens to humans and animals. Pyrethroid (PYR) insecticides have been widely used to control agricultural pests and disease vectors. Multiple reports have documented house fly resistance to PYR insecticides.

**Methods:**

In this study, we assessed the resistance levels of *M. domestica* populations collected from slaughterhouses in Riyadh, Jeddah, and Taif, Saudi Arabia, against the PYR insecticide deltamethrin (DM). We also examined the genetic mutations in the voltage-sensitive sodium channel (*Vssc*) and *P450* genes of the collected field flies and analyzed the correlation between these detected genetic mutations and the levels of DM resistance.

**Results:**

The house fly field populations showed very high levels of resistance to DM, with resistance ratio (RR) values of 625-, 256-, and 107-fold for Jeddah, Taif, and Riyadh, respectively. Three VSSC resistance alleles, kdr (T929 + 1014F), kdr-his (T929 + 1014H), and 1B (929I + 1014F), along with the susceptible allele (T929 + L1014) were identified in the Saudi house fly populations. The super-kdr allele (918 T + 1014F) and type N (D600N + M918T + L1014F) were not detected in Saudi house fly populations. Type 1B was the most dominant VSSC resistance allele, followed by kdr and kdr-his, in both field populations and the surviving flies exposed to DM. The resistance *CYP6D1v1* allele of *P450* was detected in slaughterhouse house fly populations of Riyadh, Jeddah, and Taif, with frequencies of 71%, 58%, and 60%, respectively. The *VSSC* resistance alleles exhibited a positive correlation with the resistance levels to DM; conversely, the *CYP6D1v1* displayed a negative correlation with DM resistance levels.

**Conclusions:**

In general, the Saudi house fly populations exhibited high genetic diversity, with three VSSC resistance alleles identified in slaughterhouse populations. The *Vssc* mutations appear to be the principal mechanism of DM resistance in Saudi house fly field populations. This study is the first report on the *Vssc* and *CYP6D1* mutations associated with PYR resistance in *M. domestica* field populations from Saudi Arabia.

**Graphical Abstract:**

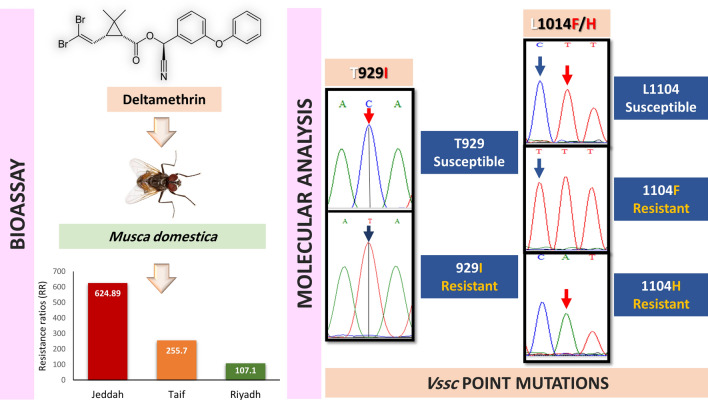

## Background

The house fly (*Musca domestica* L.) is a cosmopolitan insect pest that poses serious health risks to both humans and animals as a vector for pathogens such as viruses, bacteria, fungi, protozoa, and nematodes [1–6]. Several control techniques are employed to manage house fly populations, including sanitation, screening, waste management, utilization of biocontrol agents, and application of insecticides [6]. Although chemical control is the most effective method in managing house flies, they are resilient to chemicals and develop resistance to them [5]. Pyrethroid (PYR) insecticides, representing 30% of the global insecticide market, are employed to manage a variety of agricultural pests and disease vectors [7]. Besides their potent effectiveness against insects, the PYR insecticides have relatively low toxicity to warm-blooded vertebrates [8]. Additionally, they are degradable within 2 months, thus having a less negative impact on the environment compared with other insecticides, such as organochlorines [8, 9].

There are several reports on house fly resistance to PYR insecticides [10–14]. The two main ways by which house flies develop resistance are (1) target-site insensitivity caused by knockdown resistance (*kdr*) mutations in the voltage-sensitive sodium channel (*Vssc*) gene and (2) enzyme detoxification [4]. The former was first documented in the house fly in 1951 [15]. There are currently many pest species carrying the classic *kdr* mutations, for example the replacement of phenylalanine by a leucine at position 1014 of VSSC polypeptide, leading to the channel's insensitivity to PYR insecticides [16–19]. Numerous investigations have connected the paratype *Vssc* with the kdr and super-kdr (s-kdr) phenotypes in house flies [20, 21] and related resistance mechanisms in other insect species [22, 23]. The complete sequence of a house fly paratype *Vssc* gene was first published in 1996 and subsequently its single-nucleotide polymorphisms (SNPs), leading to amino acid substitutions in the VSSC protein, were detected in insecticide-resistant houseflies [16, 24–26].

The second way by which house flies develop resistance is through enzymatic detoxification. Detoxification mechanisms are characterized by the upregulation of enzyme production or a qualitative change in detoxifying enzymes [27]. These enzymes include cytochrome P450 (CYP)-dependent monooxygenases or non-specific esterases such as carboxylesterases [28–31]. There are 146 CYPs in the house fly, carrying out a broad range of functions including the metabolism of natural and synthetic toxic chemicals [32]. These genes confer resistance to a number of insecticide classes including organophosphates [33], PYRs [34, 35], and neonicotinoids [36]. Among the *P450* genes conferring insecticide resistance in house flies are *CYP6D1* and *CYP6D3*, located on chromosome I [37, 38]. The two genes have similar genetic structures, consisting of five exons and four introns of almost the same length, suggesting that one of these genes may have resulted from a duplication event [38].

The main objectives of the present study were to (1) evaluate the resistance levels of *M. domestica* slaughterhouse populations collected from Riyadh, Jeddah, and Taif against the PYR insecticide deltamethrin (DM), (2) investigate the genetic mutations of *Vssc* and *P450* genes in the collected field flies and their survivor counterparts after exposure to DM, and (3) determine the correlation between genetic mutations of *Vssc* and *P450* and level of DM resistance. The outcomes of the present study provide valuable information that may help in controlling house fly populations in Saudi Arabia.

## Methods

### Collection and rearing of house flies

Field populations of house flies were collected by sweep net from slaughterhouses (about 100 adult house flies/population) in different regions of Saudi Arabia, namely Riyadh (Riyadh Automated Slaughterhouse, N24.5793252166357, E46.73542482697681), Jeddah (East Jeddah Slaughterhouse, N21.53832286527901, E39.25652725404367), and Taif (Taif Municipality Ideal Slaughterhouse, N21.34799321587203, E40.45159577841662). The collected house flies (either live or preserved in 95–100% ethanol) were transported to the Insect Breeding Laboratory, Department of Plant Protection, College of Food and Agriculture Sciences, King Saud University. The flies stored in 95–100% ethanol were used for molecular analysis. The live house flies were designated as the parental generations and allowed to randomly mate. The first filial generation (F1) progenies (3–5 days old) were used for toxicity bioassays. A susceptible laboratory strain (LAB, bred since 2006) was brought from the Public Health Pests Laboratory (PHPL, Jeddah, Saudi Arabia). The LAB strain and field house flies were kept at 25 ± 2 °C, 30–40% relative humidity, and 12:12 h light–dark cycle. Adult flies were fed on a mixture of 2% milk powder and 10% sucrose. Eggs were collected from the cages and cultured in a larval medium containing wheat bran, yeast, milk powder, and water at proportions of 20:1:2:20, respectively [39].

### Insecticide

The PYR insecticide DM [(*S*)-cyano-(3-phenoxyphenyl) methyl (1*R*,3*R*)-3-(2,2-dibromoethenyl)-2,2-dimethylcyclopropane-1-carboxylate, 98.6%] (Hunan Haili Chemical Industry Co., Hannan, China) was used in this study.

### Bioassays

The topical application method was used for assessing the resistance levels in house flies towards DM according to Scott et al. [40]. A 10 mg/ml stock solution of 90.5% DM was first prepared in acetone and then diluted to at least five serially diluted concentrations (100–8000 µg/ml). Based on the preliminary dose–response results, the five DM doses for the LAB strain were 1, 2, 4, 8, and 16 ng/fly. However, the five DM doses for Riyadh, Jeddah, and Taif flies were 100, 200, 400, 800, 1600, 3200, and 6400 ng/fly; 500, 1000, 2000, 4000, and 8000 ng/fly; and 200, 500, 1000, 2000 and 4000 ng/fly, respectively. A total of 1 µl of each diluted insecticide concentration was applied on the thoracic notum of 3–5-day-old flies. For control treatments, 1 µl of acetone was applied for each fly. Four replicates, each with 20 flies, were used for each DM concentration. Flies were anesthetized by CO_2_ for 20 s before their treatments. The treated flies were maintained at 25 ± 2 °C under a 12:12 h light–dark cycle. Mortality was assessed 24 h after treatments. Flies that did not move when touched with a soft brush were scored as dead.

### *Vssc *and *P450* genotyping of field and DM-surviving house flies

Genomic DNA was extracted from individual house flies, including field and DM-surviving flies, using the QIAamp DNA Mini Kit (Qiagen, Hilden, Germany) according to the manufacturer’s protocols. The quality and quantity of DNA solutions were determined with a NanoDrop spectrophotometer (Thermo Scientific™, Waltham, MA, USA) and agarose gels according to Sambrook and Russell [41]. For polymerase chain reactions (PCRs), the concentrations of extracted DNA solutions were adjusted to 20 ng/µl and then stored at −20 ºC for further molecular work.

Three *Vssc* gene fragments were amplified by PCR in a 30 μl reaction volume containing 15 μl of 2× GoTaq Green Master Mix (Promega Corporation, Madison, WI, USA), 2 μl of 20 ng/µl DNA template, 10 μl of nuclease-free water, and 1.5 μl of 10 μM forward primer KdrDIGLongF (5′-TCGCTTCAAGGACCATGAATTACCGCGCTG-3′) and 10 μM reverse primer KdrDIGLongR (5′-CCGAAGTTGGACAAAAGCAAAGCTAAGAAAAG-3′) for the kdr fragment; 10 μM forward primer MdSCF52 (5′-GCAAAATCATGGCCCACACT-3′) and 10 μM reverse primer MdSCR3 (5′-GTTCTTTCCGAAAAGTTGCATTCC-3′) for the s-kdr fragment; and 10 μM forward primer MdSCF61 (5′-AATACGAAATGGGCGTGGAC-3′) and 10 μM reverse primer MdSCR62 (5′-CATTCTCTTCGGACATTGGTG-3′) for the type N fragment. For the *P450* fragment; 10 μM forward primer Md6D1F1 (5′-CCGTCATTTACAACGCATTAGG-3′) and 10 μM reverse primer Md6D1R2 (5′-ACCTTYTCGTGGCATTTGTC-3′) were used to amplify the fragments from studied house fly samples [11]. PCR conditions were as follows: 94 °C for 3 min, followed by 35 cycles of 94 °C for 30 s, annealing temperature according to each primer pair for 30 s and 72 °C for 60 s, and a final extension step of 5 min at 72 °C [11]. PCR products were run on 1.5% agarose gels stained with 0.5 µg/ml acridine orange. PCR products were directly sequenced, with the same primers as previously mentioned, at the Macrogen sequence facility (Macrogen, Seoul, Korea). DNA sequences were cleaned and edited manually using BioEdit (https://www.nucleics.com/DNA_sequencing_support/Trace_viewer_reviews/BioEdit/) and Geneious (https://www.geneious.com) software. The cleaned sequences were searched against the National Center for Biotechnology Information (NCBI) GenBank database to obtain their homologous counterparts. BioEdit was used to detect SNPs and assign combinations of different mutations. A total of 151 individual house flies collected from slaughterhouses in Riyadh (51), Jeddah (50), and Taif (50) were genotyped for the *Vssc* kdr (L1014F/H), s-kdr (M918T and T929I), type N (D600N), and *CYP6D1v1* sites. Additionally, 15 DM survivors from each field population were genotyped at the same four sites. For flies heterozygous at two *Vssc* sites, the resistance allele identified in DM survivors was assumed to correspond to the resistance allele observed in field flies.

### Statistical analysis

The median lethal dose (LD_50_) for DM was determined by probit analysis [42]. Significant differences between LD_50_ values were based on no overlapping 95% fiducial limits (FL) [43]. Resistance ratios (RRs) were calculated by dividing the LD_50_ value for a field population by the LD_50_ of the laboratory strain [44]. The resistance levels were categorized as described by Torres-Vila et al. [45], as follows: RR < 2 (no resistance), RR = 2–10 (low resistance), RR = 11–30 (moderate resistance), RR = 31–100 (high resistance), and RR > 100 (very high resistance). Linear regression analysis was used to assess the correlations between RRs of different populations with VSSC and *P450* mutations. All analyses were conducted using SPSS 26 software (IBM Corp., Armonk, NY, USA).

## Results

### Susceptibility of house flies to DM

Based on DM bioassay data, the LD_50_ values of adult *M. domestica* slaughterhouse populations collected from the Riyadh, Jeddah, and Taif regions in Saudi Arabia were significantly higher than those of the LAB strain (Table 1). Moreover, there were significant differences among DM LD_50_ values of the three slaughterhouse populations. The highest LD_50_ was recorded for the Jeddah population (1793 ng/fly), followed by Taif (734 ng/fly) and Riyadh (307 ng/fly). Overall, the house fly field populations displayed very high resistance to DM, where the recorded RRs were 625-, 256-, and 107-fold for the Jeddah, Taif, and Riyadh populations, respectively (Table 1).

**Table 1:** Deltamethrin median lethal dose (LD_50_) values and resistance ratios (RRs) for *Musca domestica* collected from three slaughterhouses in Saudi Arabia

Population	No.^a^	LD_50_ (ng/fly)	95% fiducial limits		Slope ± SE	*χ*^2^	*P*	RR
			Lower	Upper				
LAB	360	2.87^d^	2.43	3.44	2.35 ± 0.24	6.77	0.08	–
Jeddah	360	1793^a^	1346	2358	1.29 ± 0.19	2.29	0.52	625
Taif	360	734^b^	607	878	2.16 ± 0.21	5.09	0.17	256
Riyadh	360	307	220	408	1.17 ± 0.14	1.80	0.77	107

LD_50_ values with different superscript letters are significantly different (no overlapping 95% fiducial limits)

^a^Number of individual flies

*SE* standard error

### Molecular data

#### *Vssc* single mutations

The primer pairs targeting the kdr (L1014F/H), s-kdr (M918T and T929I), and type N (D600N) regions of the *Vssc* locus generated PCR products of 346, 138, and 140 base pairs (bp), respectively. *Vssc* sequences were deposited at GenBank under accession numbers PP586084–PP586085 and PQ800608–PQ800625 for 929 point mutations, and PP586082-PP586083 and PQ800592-PQ800607 for 1014 point mutations. The type N (D600N mutation) region showed no mutation in either the three field house fly populations or the surviving flies exposed to DM insecticide. However, kdr-L1014F/H and T929I mutations were detected in field populations and their counterpart survivors (Table 2).

**Table 2:** Frequencies of T929I and kdr-L1014F/H point mutations and their genotypes

Population		929 allele		929 genotype			1014 allele			1014 genotype					
	N	T^b^	I	T/T^b^	T/I	I/I	L^b^	F	H	L/L^b^	F/F	L/F	H/H	L/H	F/H
Riyadh	51	64 62.7%	38 37.3%	29 56.9%	6 11.7%	16 31.4%	45 45%	53 53%	2 2%	17 34%	21 42%	11 22%	1 2%	0 0	0 0
Riyadh-SV^a^	15	3 10%	27 90%	1 6.7%	1 6.7%	13 86.6%	1 3.3%	27 90%	2 6.7%	0 0	13 86.6%	1 6.7%	1 6.7%	0 0	0 0
Jeddah	50	37 37%	63 63%	11 22%	15 30%	24 48%	23 23%	75 75%	2 2%	8 16%	34 68%	7 14%	1 2%	0 0	0 0
Jeddah-SV	15	4 13.3%	26 86.7%	0 0	4 26.7%	11 73.3%	0 0	15 100%	0 0	0 0	15 100%	0 0	0 0	0 0	0 0
Taif	50	67 67%	33 33%	26 52%	15 30%	9 18%	47 49%	40 43.7%	9 7.3%	13 27%	12 23%	16 37.5%	1 2%	5 6.3%	2 4.2%
Taif-SV	15	0 0	30 100%	0 0	0 0	30 100%	0	15 100%	0	0 0	15 100%	0 0	0 0	0 0	0 0

^a^Populations ending with -SV are flies that survived after exposure to deltamethrin

^b^Susceptible allele or genotype

#### kdr-L1014F/H mutations

The two resistance mutations at the kdr 1014 position (L1014F/H) were detected in the three field populations (Table 2). However, 1014F was only detected in the surviving flies of Jeddah and Taif which survived DM treatment (Table 2). At the kdr 1014 genotype level, four genotypes (L/L, F/F, L/F, and H/H) were detected in the Riyadh population. The susceptible homozygous genotype (L/L) was represented by 17/50 of the analyzed individuals. The remaining 33 individuals carried either homozygous (21/50, F/F and 1/50, H/H) or heterozygous (11/50, L/F) resistant genotypes (Table 2). The Jeddah slaughterhouse population had the same four genotypes but with different frequencies as follows: 8/50 for L/L, 34/50 for F/F, 1/50 for H/H, and 7/50 for L/F (Table 2). However, for the Taif population, six genotypes at the kdr 1014 position were found. The homozygous genotypes were L/L (13/49), F/F (12/49), and H/H (1/49). The heterozygous genotypes were L/F (16/49), L/H (5/49), and F/H (2/49) (Table 2). For Taif fly survivors exposed to DM, the homozygous resistant genotype F/F was almost the only recovered genotype (Table 2). The survivors of the Riyadh population showed two more genotypes, namely L/F and H/H, with only one individual each (Table 2).

#### T929I mutation

The 929I resistance mutation was found in the three field house fly populations with frequencies of 37.3%, 63%, and 33% in Riyadh, Jeddah, and Taif, respectively (Table 2). For the surviving flies of Riyadh, Jeddah, and Taif, the frequencies of the 929I point mutation were 90%, 86.7%, and 100%, respectively (Table 2). The three genotypes T/T, T/I, and I/I of the T929I position were detected in the field populations with different frequencies (Table 2). In the Riyadh population, the susceptible homozygous genotype (T/T) was represented by 29/51 of the analyzed individuals. The remaining 22 individuals carried either the homozygous I/I (16/51) or the heterozygous T/I (6/51) resistant genotypes. For Riyadh’s survivors, the three genotypes I/I (13/15), T/I (1/15), and T/T (1/15) were recovered (Table 2). The Taif slaughterhouse population also had the three genotypes but with different frequencies: 26/50 for T/T, 9/50 for I/I, and 15/50 for T/I. For the survivors of the Taif population, the I/I was the only recovered genotype (Table 2). For the Jeddah slaughterhouse population, the frequency of the three genotypes was 11/50 for T/T, 24/50 for I/I, and 15/50 for T/I. However, the resistant genotypes I/I (11/15) and T/I (4/15) were recovered from Jeddah’s survivors (Table 2).

#### VSSC alleles and genotypes

**Fig. 1:**
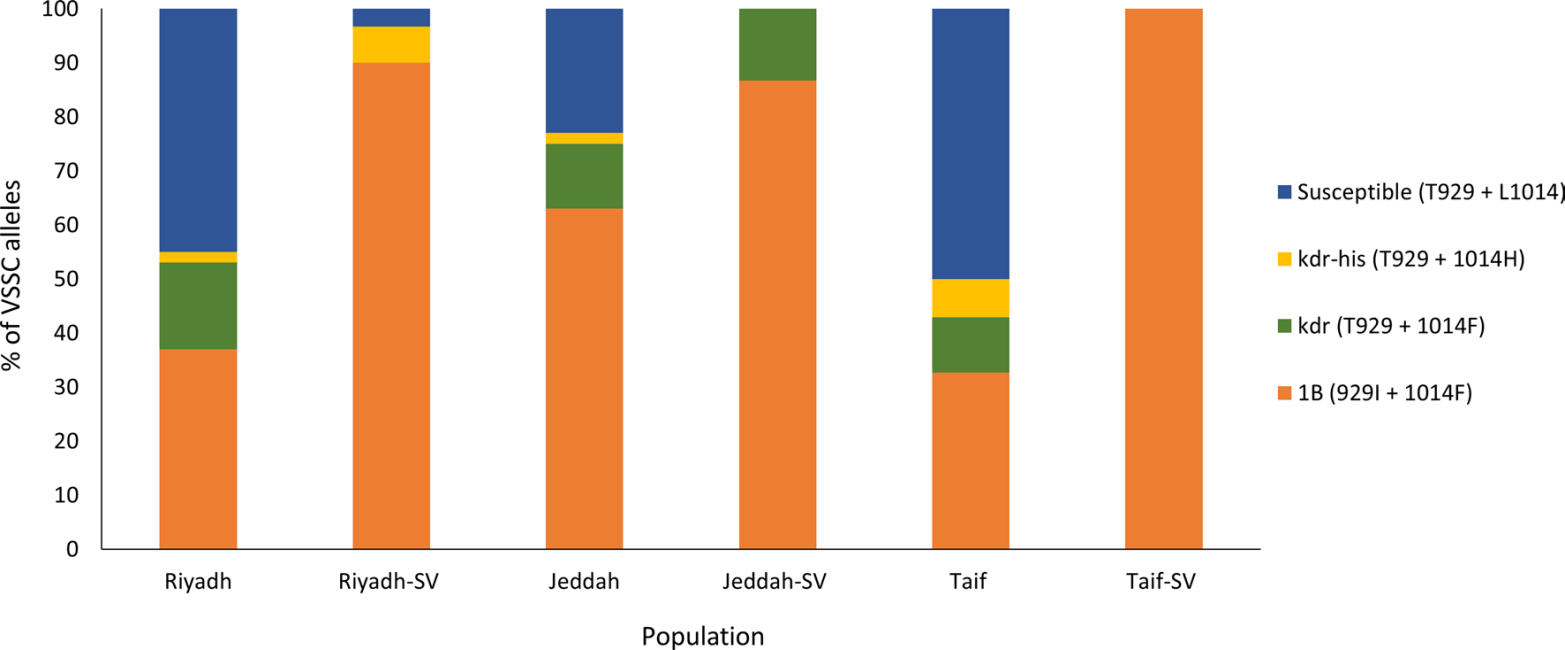
Frequencies of VSSC haplotypes recovered from field and surviving (SV) house flies collected from Saudi Arabia

The VSSC PYR resistance allele (resulting from the combination of 929 and 1014 point mutations) frequencies varied among the three slaughterhouse populations (Fig. 1). Jeddah had the highest prevalence of VSSC resistance alleles (77%) (Fig. 1). The most dominant VSSC allele was type 1B (929I + 1014F, 63%), followed by kdr (929 T + 1014F, 12%) and kdr-his (929 T + 1014H, 2%) (Fig. 1). The susceptible VSSC allele (T929 + L1014) represented 23% of the Jeddah field population (Fig. 1). Fifty percent of the Taif field population contained the susceptible allele. However, the frequencies of VSSC resistance alleles were 33% (type 1B), 10% (kdr), and 7% (kdr-his). In the Riyadh slaughterhouse population, the frequencies of VSSC resistance alleles were 37% for type 1B, 16% for kdr, and 2% for kdr-his (Fig. 1). For the surviving flies of Riyadh, two VSSC resistance alleles, type 1B (90%) and kdr-his (7%), were detected, along with the susceptible allele (3%). For Jeddah surviving flies, the two alleles type 1B (87%) and kdr (13%) were detected (Fig. 1). The type 1B was the only VSSC resistance allele detected in Taif survivors (Fig. 1).

At the VSSC genotype level, seven genotypes were recovered from the Riyadh population (Table 3). The susceptible genotype was most frequent (17/50), followed by the homozygous type 1B (16/50) (Table 3). The other homozygous VSSC genotypes were kdr (4/50) and kdr-his (1/50). The remaining 12 flies had heterozygous VSSC genotypes (Table 3). For the Jeddah field population, six genotypes were found (Table 3). The most prevalent homozygous genotype was the 1B (24/50), followed by the susceptible genotype (8/50) (Table 3). Besides the homozygous VSSC genotypes kdr (2/50) and kdr-his (1/50), there were 15 flies possessing heterozygous VSSC genotypes (Table 3). Eight genotypes were recovered from the Taif field population (Table 3). The susceptible genotype was the most frequent (15/50), followed by the resistant homozygous type 1B (9/50) and Kdr-his (1/50) genotypes (Table 3). The remaining 24 flies of Taif had heterozygous VSSC genotypes (Table 3). The VSSC genotypes recovered from surviving flies of Riyadh were the homozygous genotypes type 1B (13/15) and kdr (1/15) as well as the heterozygous type 1B/S genotype (Table 3). The two genotypes recovered from the Jeddah surviving flies were the homozygous type 1B (11/15) and the heterozygous type 1B/kdr genotype (4/15) (Table 3). The homozygous genotype type 1B (15/15) was the only genotype detected in surviving flies of Taif (Table 3).

**Table 3:** VSSC genotypes recovered from field-collected house flies and those which survived deltamethrin treatment

VSSC genotype	929 T/I	1014 L/F/H	Riyadh	Jeddah	Taif	Riyadh-SV^a^	Jeddah-SV	Taif-SV	Total
1B	I/I	F/F	16	24	9	13	11	15	88
1B/kdr	T/I	F/F	1	8	3	0	4	0	16
kdr-his	T/T	H/H	1	1	1	1	0	0	4
kdr	T/T	F/F	4	2	0	0	0	0	6
S^b^	T/T	L/L	17	8	15	0	0	0	40
kdr/S	T/T	L/F	7	0	7	0	0	0	14
kdr-his/S	T/T	L/H	0	0	3	0	0	0	3
1B/S	T/I	L/F	4	7	9	1	0	0	21
1B/kdr-his	T/I	F/H	0	0	2	0	0	0	2
Total			50	50	49	15	15	15	194

^a^Populations ending with -SV are flies that survived after exposure to deltamethrin

^b^S susceptible

#### *CYP6D1v1* genotyping

**Fig. 2:**
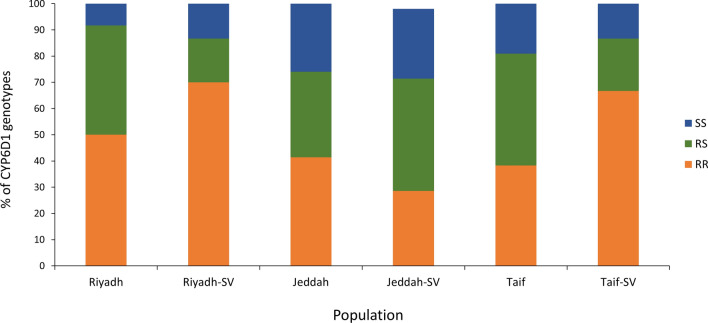
Frequencies of *CYP6D1* genotypes recovered from field house flies collected from Saudi Arabia and their surviving (SV) counterparts exposed to deltamethrin. SS, homozygous susceptible, RS, heterozygous resistant, RR, homozygous resistant

The CYP6D primers generated PCR amplicons of either 424 bp (susceptible allele, accession number PP586081) or 439 bp (resistance allele, accession number PP112328) from individual house flies of field populations and their survivors exposed to DM. The studied field populations of Saudi Arabia had the resistance allele *CYP6D1v1*, with frequencies of 71%, 58%, and 60% in Riyadh, Jeddah, and Taif, respectively. However, the surviving flies of Taif had a greater frequency of the *CYP6D1v1* allele (77%) than their counterparts in Riyadh and Jeddah (70% and 50%, respectively). According to *CYP6D1v1* genotype (RR + RS) frequencies, the Riyadh field population showed the highest frequency (92%), followed by Taif (81%) and then Jeddah (74%). The percentages of the *CYP6D1v1* genotype in surviving flies of Riyadh, Taif, and Jeddah were 87%, 87%, and 71%, respectively (Fig. 2).

#### Correlation of *Vssc* and *P450* mutations with level of resistance to DM

The linear regression analyses of DM resistance levels with T929I and kdr-L1014F/H mutations of VSSC showed strong positive correlations of RR values with both individual and combined mutations (*r* ≥ 0.985, Table 4). However, the *P450* mutation was negatively correlated with DM resistance levels in the three slaughterhouse populations (*r* = −0.88, Table 4). When the *Vssc* and *P450* were combined, there was a positive correlation between the point mutations and DM resistance (*r* = 0.89, Table 4).

**Table 4:** Correlation between the percentage of VSSC and *Cyp6d1v1* resistance mutations and level of resistance to deltamethrin in three house fly populations collected from Saudi Arabia

Population	RR^b^	VSSC			*Cyp6d1v1*	VSSC + *Cyp6d1v1*
		929	1014	929 + 1014		
Taif	256	50%	69%	74%	79%	91%
Jeddah	625	78%	84%	84%	74%	96%
Riyadh	107	43%	67%	67%	92%	92%
*r*^a^		0.996	0.985	0.990	−0.881	0.890
*P*-value		0.059	0.11	0.076	0.313	0.301

^a^Correlation coefficient

^b^RR deltamethrin resistance ratio for house fly field populations

## Discussion

PYR insecticides, including DM, have been widely used to control numerous public health pests worldwide because of their high insecticidal efficacy at low doses, rapid knockdown effects, and relatively low toxicity for humans [46]. However, the intensive use of PYR insecticides in pest control operations has resulted in the progressive development of PYR resistance in different insect species [4, 26, 47, 48]. In the present study, bioassay was used to determine the DM resistance levels in house flies collected from three locations in Saudi Arabia. The field-collected house flies showed very high levels of DM resistance, with RR values of 625-, 256-, and 107-fold for the Jeddah, Taif, and Riyadh populations, respectively. DM resistance in house flies has been recorded in several countries worldwide, including the USA [49], Slovak Republic [50], Turkey [51], Pakistan [10], China [12], and Iran [52]. The Jeddah field population showed the highest level of DM resistance, followed by the Taif and Riyadh populations. Although the Riyadh population showed the lowest level of DM resistance (RR = 107), this resistance level is significantly higher than the levels previously recorded by Al-Zahrani et al. [53] (RR = 10.9), who collected flies from the same slaughterhouse in Riyadh city [53]. Consistent with our results, Al-Hussein [54] reported very high resistance levels to DM (RRs > 100-fold) in house fly populations collected from different slaughterhouses in Riyadh city [54]. However, house fly populations collected from dairy farms around Riyadh showed low to moderate DM resistance levels (2–21) [55], which may be attributed to the different insecticide exposure scenarios in dairy farms compared to slaughterhouses. Other dipteran species have also exhibited high resistance levels to DM in Riyadh city, such as *Culex pipiens* (RRs = 161–168) [56], which may indicate the widespread use of DM or other PYR insecticides in Riyadh city. The highest resistance against DM in this study was recorded for the Jeddah population (RR = 625). Asid et al. [57] reported a lower ratio of resistance (4.1-fold) to DM in house fly populations collected from the same slaughterhouse in Jeddah, indicating a 156-fold increase in house fly resistance over 8 years [57]. This remarkable increase in DM resistance points to the high rate of selection pressure through the continuous application of PYR insecticides, including DM, for house fly control operations in Jeddah slaughterhouses. In fact, 36 registered DM formulations, alone or in mixtures with other insecticides, are currently used for controlling public health pests, including house flies, in Saudi Arabia [58].

The genetic basis for developing resistance in house flies depends on two major mechanisms, namely target-site insensitivity to insecticides and enzyme detoxification of them. The common target-site insensitivity to PYR insecticides occurs through knockdown mutations in the *Vssc* gene [4]. The most common kdr alleles leading to amino acid substitutions in the VSSC protein include kdr (L1014F), kdr-his (L1014H), s-kdr (M918T + L1014F), 1B (T929I + L1014F), and N type (D600N + M918T + L1014F) [59, 60]. In our study, we detected amino acid substitutions in two positions (T929I and L1014H/F) of the VSSC protein giving rise to resistance in Saudi house fly field populations. The frequencies of amino acid substitutions associated with insecticide resistance varied among Saudi populations, with higher frequencies in the Jeddah population, which showed a very high level of DM resistance, with an RR value of 625-fold. The type N mutation (D600N mutation) was not detected in the three Saudi field house fly populations or the surviving flies exposed to DM insecticide. The type N mutation was first reported in resistant house fly individuals with the VSSC allele of D600N + M918T + L1014F collected from Kansas, USA [61]. However, the D600N mutation has not previously been linked to PYR resistance in any arthropods [62]. Moreover, this mutation was only reported in house flies in the USA [60, 61].

In our study, three VSSC resistance alleles (1B, kdr, and kdr-his) were identified in Saudi slaughterhouse house fly populations. The VSSC susceptible allele (T929 + L1014) was also recovered. The frequency of the VSSC PYR resistance alleles differed among the three slaughterhouse populations, with the highest frequency in Jeddah (77%), followed by Riyadh (55%) and Taif (50%). These VSSC resistance alleles were present in the three populations. Type 1B (929I + 1014F) was the most dominant VSSC resistance allele in the three slaughterhouse populations and their surviving counterparts exposed to DM. Kasai et al. proposed that the VSSC mutant alleles confer levels of PYR resistance as follows: kdr-his < kdr < type N ≤ s-kdr ≤ 1B [61]. Consequently, type 1B confers the highest resistance to DM [11, 17, 61, 62]. Moreover, the type 1B VSSC allele has only been detected in the USA [11, 60–62] and United Arab Emirates (UAE) [63] house fly populations. Sun et al. (2017) found high levels of resistance (up to 1000-fold RR) to DM in house fly populations in Kansas, USA, which could be due to the presence of the type 1B VSSC allele [62].

The kdr (T929 + 1014F) was the second most frequent VSSC resistance allele recovered from three Saudi slaughterhouse populations. In addition, the surviving flies of Jeddah had this allele. The kdr allele had the first detected point mutation of VSSC polypeptide that offered resistance to PYR insecticides [15]. The kdr allele had a worldwide distribution, having been recovered from house fly populations in at least three continents: Europe, North America, and Asia [9, 11, 60, 64–66]. For example, resistant house flies carrying the kdr allele were found in populations collected from the UK, Denmark, Italy, and Turkey [9, 16, 64, 66]. Moreover, house flies with the kdr allele were collected from open fields in Iran, China, and Pakistan [4, 52, 67]. The kdr allele was also detected in 11 states of the USA [59].

The third most frequent VSSC allele in the three Saudi house fly populations was the kdr-his, which was also found in the surviving flies from Riyadh. Previous studies have shown that kdr-his is the second most dominant VSSC resistance allele in house fly field populations worldwide [9, 11, 60, 68, 69]. The kdr-his was reportedly the most common allele in house fly populations in four states of the USA [59].

The s-kdr allele (918 T + 1014F) was not detected in the three Saudi field house fly populations or their surviving counterparts exposed to DM insecticide. The s-kdr allele has been found in house fly populations in many countries, including the UK, USA, Turkey, and Italy [9, 16, 40, 68]. Among the different resistance VSSC alleles, the s-kdr has the highest fitness cost [11, 70], which may explain why the Saudi populations do not have this resistance VSSC allele. Moreover, multi-halogenated benzyl PYRs, including DM, are commonly used for controlling house flies in Saudi Arabia, and these PYRs have shown some toxicity on flies with s-kdr mutations [61]. In our study, we also detected the heterozygote T929I + L1014F in both the Riyadh field population and its DM survivors, suggesting that this genotype is most likely DM-resistant. However, Kasai et al. [61] detected the same genotype in the house fly field population collected from New York, USA [59], and considered as a susceptible heterozygote VSSC genotype.

The second insecticide resistance mechanism in insects involves metabolic detoxification that is achieved through different mechanisms, for example, overexpression of specific genes such as *P450* [71]. The overexpression of the *CYP6D1v1* gene is caused by a 15-bp sequence that is inserted into the 5′ promoter region [72]. The transcriptional repressor mdGfi-1 binds tenfold less to the mutated *CYP6D1* promoter with the 15-bp insert, compared with the normal promoter of susceptible flies, leading to increased transcription of the gene [71, 73]. Pan et al. [67] showed that PYR resistance mediated by *CYP6D1* overexpression is a widespread resistance mechanism in house flies [67]. In our study, the resistance allele *P450 CYP6D1v1* was detected in Saudi house fly populations with frequencies of 71%, 58%, and 60% in Riyadh, Jeddah, and Taif, respectively. However, the surviving flies of Taif had a greater frequency of the *CYP6D1v1* allele (77%) than their counterparts in Riyadh and Jeddah (70% and 50%, respectively). Although VSSC genotyping correlated well with the DM resistance levels, the *CYP6D1v1* was negatively correlated with the DM resistance levels in the three slaughterhouse populations. These results agree with Freeman et al. [11], who reported that VSSC genotyping correlated well with relative permethrin resistance levels rather than *CYP6D1v1* [11]. The *CYP6D1v1* mutation has been detected in many field house fly populations, including the USA, Turkey, and China [68, 74–76]

## Conclusions

Based on the present study, we found that field house fly populations showed very high resistance levels to the PYR insecticide DM and that resistance was relatively widespread in Saudi Arabia. Moreover, house fly populations showed high diversity, where three VSSC resistance alleles were recovered from field populations. The *Vssc* point mutations were associated with DM resistance in the Saudi house fly field populations. However, the negative correlation of the *P450* mutation with DM resistance may suggest that this mutation does not play a key role in resistance to DM in the studied population. Based on the obtained data, it is recommended that PYRs used in the house fly control programs in Saudi Arabia be replaced or rotated with other insecticide groups targeting sites other than the *Vssc* locus.

## Data Availability

Nucleotide sequences generated during the current study are deposited in GenBank (https://www.ncbi.nlm.nih.gov/genbank/) under accession numbers PP112328, PP586081–PP586085, and PQ800592–PQ800625.

## Abbreviations

DM: Deltamethrin
*kdr*: Knockdown resistance gene
PYR: Pyrethroid
RR: Resistance ratio
*Vssc**/*VSSC: Voltage-sensitive sodium channel gene/protein
